# TRPM8, a Versatile Channel in Human Sperm

**DOI:** 10.1371/journal.pone.0006095

**Published:** 2009-06-30

**Authors:** Gerardo A. De Blas, Alberto Darszon, Ana Y. Ocampo, Carmen J. Serrano, Laura E. Castellano, Enrique O. Hernández-González, Mayel Chirinos, Fernando Larrea, Carmen Beltrán, Claudia L. Treviño

**Affiliations:** 1 Departmento de Genética del Desarrollo y Fisiología Molecular, Instituto de Biotecnología, Universidad Nacional Autónoma de México (UNAM), Cuernavaca, Morelos, México; 2 Unidad de Investigación Médica IMSS, Zacatecas, Zacatecas, México; 3 Departamento de Ciencias Aplicadas al Trabajo, División de Ciencias de la Salud, Universidad de Guanajuato León, Guanajuato, México; 4 Departmento de Biología Celular, CINVESTAV-IPN, México Distrito Federal, México; 5 Departamento de Biología de la Reproducción, Instituto Nacional de Ciencias Médicas y Nutrición Salvador Zubirán, Distrito Federal, México; Sun Yat-Sen University, China

## Abstract

**Background:**

The transient receptor potential channel (TRP) family includes more than 30 proteins; they participate in various Ca^2+^ dependent processes. TRPs are functionally diverse involving thermal, chemical and mechanical transducers which modulate the concentration of intracellular Ca^2+^ ([Ca^2+^]i). Ca^2+^ triggers and/or regulates principal sperm functions during fertilization such as motility, capacitation and the acrosome reaction. Nevertheless, the presence of the TRPM subfamily in sperm has not been explored.

**Principal Findings:**

Here we document with RT-PCR, western blot and immunocitochemistry analysis the presence of TRPM8 in human sperm. We also examined the participation of this channel in sperm function using specific agonists (menthol and temperature) and antagonists (BCTC and capsazepine). Computer-aided sperm analysis revealed that menthol did not significantly alter human sperm motility. In contrast, menthol induced the acrosome reaction in human sperm. This induction was inhibited about 70% by capsazepine (20 µM) and 80% by BCTC (1.6 µM). Activation of TRPM8 either by temperature or menthol induced [Ca^2+^]i increases in human sperm measured by fluorescence in populations or individual sperm cells, effect that was also inhibited by capsazepine (20 µM) and BCTC (1.6 µM). However, the progesterone and ZP3-induced acrosome reaction was not inhibited by capsazepine or BCTC, suggesting that TRPM8 activation triggers this process by a different signaling pathway.

**Conclusions:**

This is the first report dealing with the presence of a thermo sensitive channel (TRPM8) in human sperm. This channel could be involved in cell signaling events such as thermotaxis or chemotaxis.

## Introduction

Fertilization is a complex event that requires sperm-egg communication and involves several sequential steps to finally generate a new organism. Signaling cascades involving intracellular Ca^2+^ ([Ca^2+^]i) changes regulate sperm fundamental functions such as maturation, swimming, finding and fusing with the egg [1].

Cells utilize different mechanisms to regulate their [Ca^2+^]i. Voltage dependent Ca^2+^ channels (Ca_v_s) are important players during signaling and in fact several Ca_v_s have been detected in sperm from different species [2]. The TRP superfamily also contributes significantly to Ca^2+^ signaling in different cell types. TRPs are classified into seven groups (TRPC, TRPV, TRPM, TRPA, TRPP, TRPN and TRPML) displaying a large variety of gating and regulatory mechanisms, and therefore participate in diverse cellular processes. In sperm, however, only the TRPC subfamily has been explored and indeed several of its members have been detected [3]–[5]. They are heterogeneously distributed in these cells, suggesting their participation in distinct functions at particular sperm locations. For example, TRPC2 is present in the head of mouse sperm where it has been proposed to participate in the acrosome reaction (AR) [3]. Searching for the egg, sperm encounter complex changes in media composition, viscosity and temperature. We reasoned that other members of the TRP family may be present in sperm to contend with the variety of signaling demands required for fertilization. In particular, TRPM channels are good candidates since they participate in sensory physiology, both at the cell and whole organism level. TRPM members are responsible for sensing, among other stimuli, temperature, osmolarity, voltage and pH [6]. Importantly, these channels are often regulated by more than one stimulus and thus regarded as signal integrators. This particular feature is presumably essential for sperm during their adventurous journey towards the egg. Interestingly, olfactory receptors have been already reported in sperm as possible transducers of sperm-egg communication, although the natural ligand (s) is (are) still a mystery [7].

## Materials and Methods

### Ethics

The study was approved by the Bioethics Committee at the Biotechnology Institute from the National Autonomous University of Mexico. All participants gave written informed consent.

### Materials

Menthol and fluorescein-isothiocyanate-coupled *Pisum sativum agglutinin* lectin (FITC-PSA) were purchased from Sigma Chemical Co. (St. Louis, MO). BCTC (N-(4-t-Butylphenyl)-4-(3-Chloropyridin-2-yl) tetrahydropyrazine-1(2H)-carboxamide) and capsazepine were from BIOMOL Research Lab (Plymouth, PA). Ionomycin was from Alomone (Jerusalem, Israel). Fluo-3 AM and Alexa488 were from Invitrogen (Carlsbad, CA). All other chemicals were of reagent grade. Stock solutions in DMSO were prepared for each compound and aliquots were stored at −20°C. Anti-TRPM8 were from Gene Tex (Irvine, CA) and Santa Cruz Biotechnology (Santa Cruz, CA). Oligonucleotides were from Qiagen (Valencia, CA) with the following sequences, sense: 3′ATCTATGAGCCCTACCTG 5′, antisense: 3′ AATAACATCAAGTAAGGCTG 5′ with a Tm = 51°C and an expected fragment size of 611 bp. Human recombinant ZP3 was obtained as previously described [8]

### Cell preparation

Ejaculates were obtained by masturbation from healthy donors after at least 48 hours of sexual abstinence. Only samples that fulfill the World Health Organization parameters were selected for experiments. Highly motile sperm were recovered?after a swim-up separation for 1 h in Ham's F-10 medium supplemented with 5 mg/ml of bovine serum albumin (only for capacitation conditions) at 37°C in an atmosphere of 5% CO_2_/95% air. Cell concentration was then adjusted to 5−10×10^6^ sperm/ml with Ham's F-10 and incubation was continued (capacitating conditions) for at least 4 h -when capacitation was required-.

### RT-PCR

Total RNA was isolated from human semen (without abstinence) using TRIzol reagent (Invitrogen) and cDNA was synthesized using the Superscript first strand synthesis system (Invitrogen) according to the manufacturer's instructions. Specific primers for TRPM8 channel were designed based on coding sequences. PCR programs included 35 cycles of amplification (94°C for 1 min, 55°C for 1 min, and 72°C for 30 s), and a final extension at 72°C for 5 minutes. The amplified PCR fragments were separated on 1% agarose gels, purified and sequenced. Sequence identities were established by searching the databases using NCBI- BLAST programs. The sequence has been deposited in GenBank (Accession Number FJ895300).

### Indirect immunofluorescence

Human sperm were fixed in 4% formaldehyde/PBS, washed 3×5 min with PBS and then air-dried on glass slides. Cells were permeabilized with 0.1% Triton X-100 in PBS for 10 min, washed 3×5 min with PBS and incubated for 30 min in a 2% BSA blocking solution. Samples were then incubated overnight at room temperature with primary antibodies at a 1∶100 dilution. After washing 3×5 min with PBS, samples were incubated for 1 h at room temperature with a secondary antibody conjugated to Alexa 488 (Molecular Probes Inc., Jerusalem). Sperm immunolocalization of TRPM8 channels was performed by confocal fluorescence microscopy as previously described [4].

### Western blot experiments

Human washed (twice, 1000 xg/5 min in Ham's F-10 medium, 4°C) sperm and mouse brain homogenates were resuspended in sample buffer (2% SDS, 10% glycerol, 1.6 mM EDTA, 1% 2-mercaptoethanol, 0.2 µg/ml bromophenol blue, 100 mM Tris pH 6.8) and boiled for 10 min. Samples were centrifuged at 10,000 xg for 15 min. After centrifugation, the supernatants were collected and 2-mercaptoethanol was added to a final concentration of 5% (v/v), the samples were boiled for additional 5 min, and then subjected to 10% SDS-PAGE. Proteins were electro-transferred (0.5 A/60 min in 190 mM glycine, 25 mM Trisma base, pH 8.6) to Immobilon P (Millipore) membranes (pre-treated according to the manufacturer's instructions) in a semi-dry transfer cell (Bio-Rad). After blocking with 1.5% fat free milk they were incubated with anti-TRPM8 (1∶250) (Gene Tex or Santa Cruz Biotecnhology, as indicated in the figure legend) and developed with the appropriate secondary antibody conjugated to horseradish peroxidase (HRP) (Santa Cruz Biotechnology) and the SuperSignal West Pico chemiluminescent substrate detection of HRP (Pierce Protein Research Products) according to the manufacturer's instructions.

### Evaluation of human sperm motility

Motile cells were adjusted to a concentration of 8−12×10^6^ cells/ml. Video-recording of 15–20 phase contrast microscopy fields of sperm samples were made over 1–2 min. For each condition, ∼100 sperm were tracked and analyzed with the Hobson-Tracker computer-assisted semen analysis (CASA) system. Thirty frames were recorded at a frame rate of 60 Hz. The following sperm motility parameters were measured: 1) % motility, 2) % active motility, 3) % hyperactive motility, 3) progressive velocity (VSL), 4) path velocity (VAP), 5) curvilinear velocity (VCL), 6) amplitude of lateral head displacement (ALH), 7) linearity (LIN = VSL/VCL×100%) and 8) straightness (STR = VAP/VCL×100%). Only selected parameters are shown in Supplementary Figure S1.

### Acrosome reaction assays

Capacitated sperm were divided in 30–50-µl aliquots. Each aliquot was treated as described in the figure legends. AR was induced with the indicated concentrations of menthol, progesterone or recombinant human ZP3 [8] in the presence or absence of TRPM8 inhibitors. The different reagents were added successively without washing. All incubations were carried out at 37°C. At the end of the assay, 10 µl of each sample was spotted on slides and fixed/permeabilized in ice-cold methanol. Acrosomal status was evaluated by staining with FITC-PSA according to Mendoza C et al., [9]. Briefly, spermatozoa displaying an intact acrosome are strongly labeled with the fluorescent lectin at the acrosomal region. Cells that have undergone acrosomal exocytosis show no labeling in this region or limited labeling in the posterior edge of the granule (equatorial staining). At least 200 cells were counted per experimental condition.

### Estimation of the acrosomal reaction index (ARI)

Negative (no stimulation) and positive (10 µM ionomycin) controls were included in all experiments. For each experiment, ARIs were calculated by subtracting the number of reacted spermatozoa in the negative control (spontaneous AR range was 16%–23%) from all values, the resulting values were expressed as a percentage of the AR observed in the positive control (the maximum AR range observed with Ca^2+^ ionophore was 54%–60%).

### Sperm [Ca^2+^]_i_ measurements

Motile sperm were adjusted to a concentration of 5−10×10^6^ cells/ml and incubated with Fluo-3 AM (2 µM) and 0.02% pluronic acid at 37°C during 30 min. Cells were washed once with human sperm medium (HSM) in mM: 120 NaCl, 4 KCl, 2 CaCl_2_, 15 NaHCO_3_, 1 MgCl_2_, 10 HEPES, 5 D-Glucose, 1 Sodium pyruvate, 10 L(+) lactate acid adjusted to pH 7.4. [Ca^2+^]i changes were measured in cell population or single cell experiments in HSM with 2 mM CaCl_2_ final unless otherwise specified.

#### Cell population measurements

Cells (5−10×10^6^ cells/ml) were placed in a 600 µl cuvette with continuous stirring. Fluorescence intensity was measured with an Aminco 8000 spectrofluorimeter (λ_Ex_ = 505, λ_Em_ = 525). Data were collected during 300 s at a frequency of 0.5 Hz.

#### Single cell measurements

Sperm were immobilized on poly-L-lysine (0.1% w/v) coated round coverslips (poly-L-lysine drops were air dried followed by one rinse with water) which were mounted on a chamber and placed on the stage of an inverted Nikon Diaphot 300 microscope. Fluorescence illumination was supplied by a Luxeon V Star Lambertian Cyan LED part # LXHL-LE5C (Lumileds Lighting LLC, San Jose) attached to a custom-built stroboscopic control box. The LED was mounted into a FlashCube40 assembly with dichroic mirror M40-DC400 (Rapp Opto Electronic, Hamburg) (bandwidths: excitation 450–490 nm, dichroic mirror 505 nm and emission 520–560 nm). LED output was synchronized to the Exposure Out signal of a Cool Snap CCD camera via the control box to produce a single flash of 1 ms duration per individual exposure. The camera exposure time was set equivalent to flash duration (1 ms). Images were collected every 500 ms using IQ software (Andor Bioimaging, Belfast) and a Plan Apo 60x/1.40 -oil- Nikon objective.

#### Temperature stimulation

Coverslips with cells attached were placed in a micro-incubator chamber and then filled with 1 ml of the appropriate medium (HSM) previously warmed at 37°C. The temperature was then lowered to 25°C and data was collected while lowering the temperature from 25 to 13°C with a Bipolar Temperature Controller (Harvard Bioscience Company, Massachusetts, USA).

### Image processing

Images were processed using IQ software (Andor Bioimaging, Belfast) and Image J (National Institutes of Health, http://rsb.info.nih.gov/ij/). Background subtraction was performed using a region of interest placed as close as possible to the sperm of interest. Any incompletely adhered sperm that moved during the course of any experiment were discarded. Fluorescence measurements in individual sperm were made by manually drawing a region of interest around the head for each sperm or a group of cells. Results are presented as pseudo color [Ca^2+^]i images as indicated on the individual figures.

### Data processing

Fluorescence data were processed offline using the IQ software (Andor Bioimaging, NC) and self written Igor Programs. A lasso was drawn around the head of each spermatozoon in the field of view. Cells were removed from analysis if the spermatozoon moved out of the lasso. Raw intensity values were imported into Microsoft Excel and normalized using the next equations:



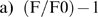



Where F is fluorescence intensity at time t and F0 is the mean of F taken during the control period. The total series of (F/F0) – 1 was then plotted vs. time.



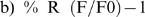



Where % R is the relative fluorescence normalized to the fluorescence obtained after the menthol or progesterone addition.

## Results

Ca^2+^ signaling regulates various important sperm functions. Although several Ca_v_s and TRPC channels have been detected in sperm from different species, little is known about the presence and role of TRPM members of the TRP family in human sperm. In other cell types, these channels sense and respond to different environmental cues such as temperature, osmolarity, pH, mechanical stress, etc., all of which are present during sperm transit through the female tract.

**Figure 1:**
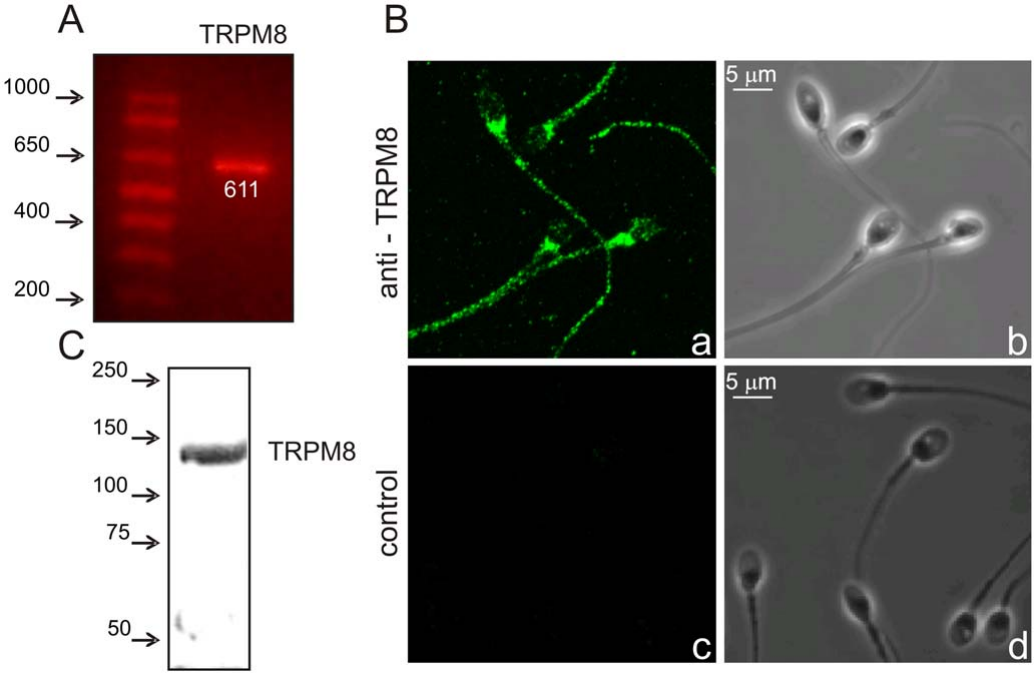
TRPM8 channels are present in human sperm. A) RT-PCR showing a TRPM8 fragment amplified from human semen. Total RNA was extracted from human semen and cDNA was prepared. Specific oligonucleotides were used for TRPM8. Identity of the expected size (in white numbers) fragment was confirmed by DNA sequencing. B) Immunocytochemistry showing the presence of TRPM8 in heads and flagella of human sperm. Left panels show the confocal fluorescent images with pseudocolor and right panels show their corresponding phase contrast images. C) Western blot experiments showing TRPM8 reactivity (∼130 kDa) in sperm lysates (anti TRPM8 from Gene Tex). n≥3.

Considering the availability, at the time, of specific antibodies, agonists and antagonists against TRPM8 channel, we explored its presence and functional role in human sperm. First, we detected mRNA for TRPM8 in cDNA prepared from human semen. Figure 1A shows RT-PCR experiments using specific oligonucleotides probes for this channel. A fragment of the appropriate size was detected and TRPM8 identity was confirmed by DNA sequencing of the amplified fragment. Negative control reactions without reverse transcriptase did not amplify any fragment (not shown). Primers were designed to amplify a fragment that could discriminate from genomic DNA contamination (coding sequence corresponding to two different exons).

Using anti-TRPM8 we observed the protein in the head (irregular staining) and in the flagella (punctuate pattern) of human sperm (Fig. 1B, panel a, b). Control experiments incubated only with secondary antibody showed no signal (Fig. 1B, panel c, d). Western blot analysis from whole cell homogenates confirmed the presence of TRPM8, a single band with the expected molecular weight (∼130 kDa) was detected (Fig. 1C). We used mouse brain homogenates as a positive control for the anti-TRPM8. In this tissue we detected a band of the same molecular weight as that seen in human sperm homogenates (Supplementary Figure S2).

As TRPM8 was immunologically found uniformly distributed along the sperm flagella, we evaluated if menthol, the principal TRPM8 agonist, influenced the motility pattern of non-capacitated and capacitated sperm using a CASA system. Even at the highest menthol concentration used (1 mM) we did not observe significant changes in the main motility parameters of non-capacitated or capacitated sperm (Supplementary Figure S1). A more detailed analysis of the various motility parameters (flagellar curvature, bending angle, frequency of beating, chemoattraction, etc.) would be required to completely rule out TRPM8 participation on this sperm function.

**Figure 2:**
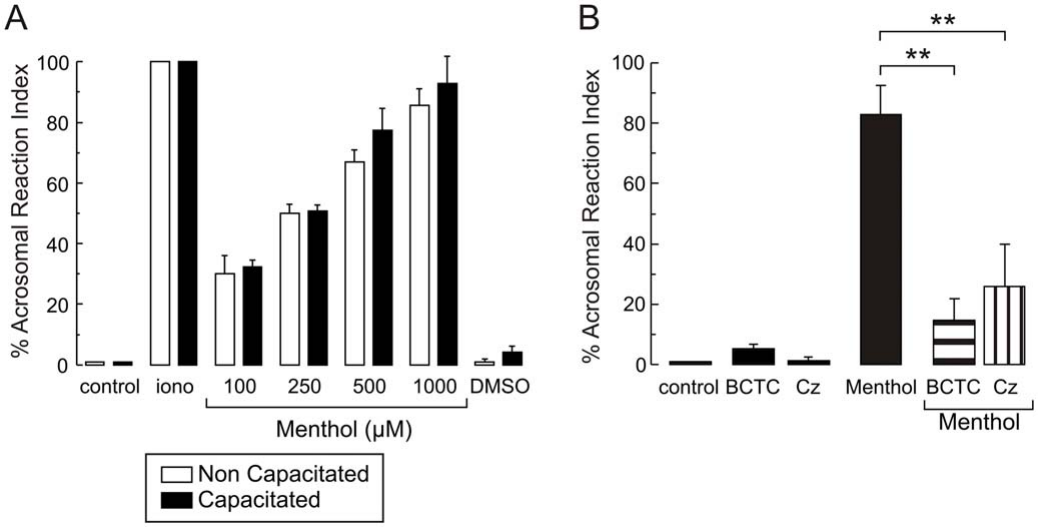
A) Menthol induces the AR in human sperm in a dose dependent manner independently of capacitation. Human sperm were separated by swim-up and capacitated (black bars) or not (white bars) during 5 hours. The % Acrosomal Reaction Index (see methods) mean values±SD from control sperm (spontaneous) and treated with various menthol concentrations, ionomycin (iono) and DMSO (the solvent) are shown. B) TRPM8 antagonists block the AR induced by menthol. After swim-up separation, human sperm were capacitated for at least 5 hours. The AR was induced with 500 µM menthol in the presence or absence of BCTC (1.6 µM) or capsazepine (Cz, 20 µM). Both inhibitors blocked the menthol induced AR [** (P< = 0.01), n≥5]. Controls using the inhibitors alone or solvent alone (DMSO) are also shown (black bars).

In other cell types, TRPM8 main function is to conduct Ca^2+^ in response to low temperature and to agonists like menthol [10]. As we also detected TRPM8 in the sperm head, we next tested whether menthol could induce the AR. Figure 2A shows that menthol induces the AR in a dose dependent manner with an apparent IC_50_ = 300 µM and also illustrates that AR induction is independent of capacitation. BCTC and capsazepine have been used as specific antagonists of TRPM8 channels; if menthol induces the AR via activation of TRPM8 channels, we hypothesized that these two drugs should block the menthol-induction of the AR. This was indeed the case; BCTC (1.6 µM) and capsazepine (20 µM) inhibited the AR by 80% and 70% respectively (Fig. 2B). Higher concentrations of BCTC or capsazepine were not tested as they can affect other channels [11].

**Figure 3:**
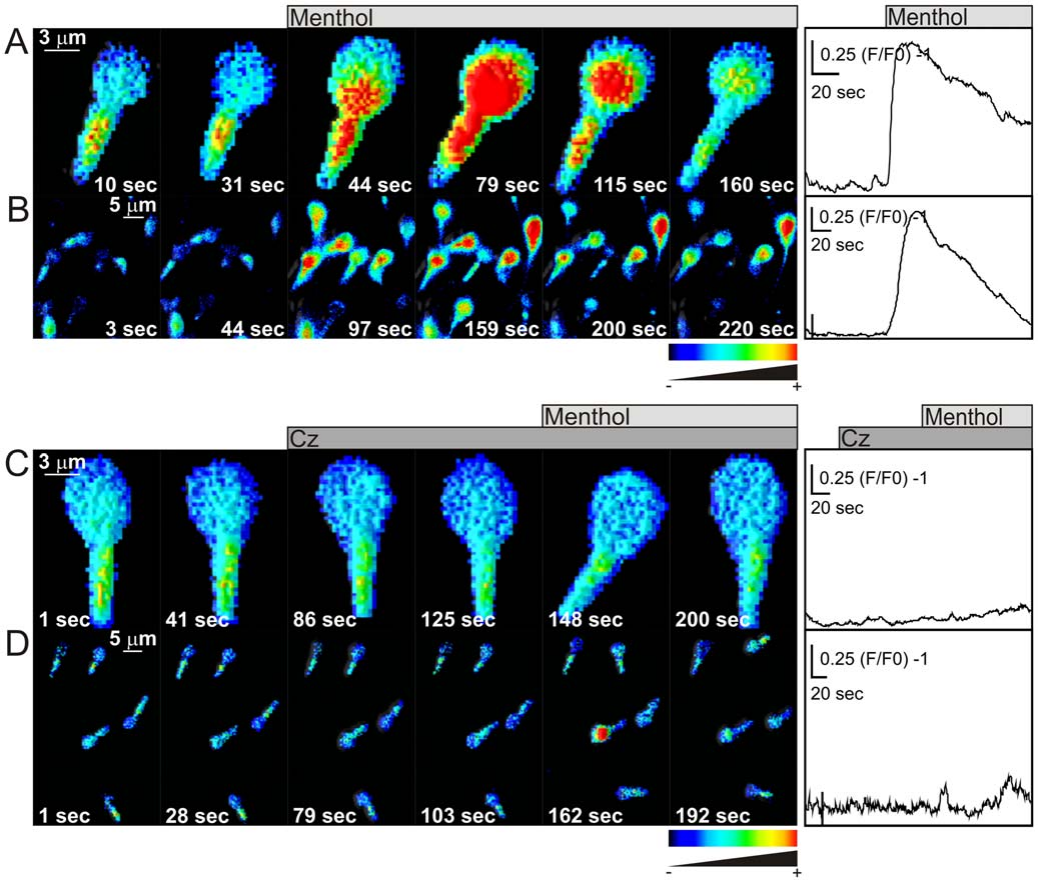
Menthol causes a capsazepine sensitive [Ca^2+^]i increase in human sperm. Human sperm were loaded with the fluorescent Ca^2+^ indicator Fluo3-AM (2 µM) and the fluorescence intensity visualized before and after menthol addition as described in methods. Representative single cell (A and C) and group of cells (B and D) spatio-temporal [Ca^2+^]i changes after adding menthol (500 µM, indicated with the light gray bar) in the absence (A and B) and presence of 20 µM Cz (C and D, indicated with the dark gray bar). The panels to the right illustrate representative traces showing the fluorescence change after addition of menthol (light gray bar), in the absence or presence of capsazepine (dark gray bar). The time frame is indicated in each panel. Scales indicate (F/F0) -1 vs time (sec). Note: ∼50% of cells responded to menthol. Color coding: black (−) to red (+) indicates low to high [Ca^2+^]i. n≥3.

Most AR inducing agents cause an elevation of [Ca^2+^]i [12] and TRPM8 activation should be accompanied by [Ca^2+^]i increases. Figure 3 shows representative [Ca^2+^]i images of Fluo3 loaded sperm individually (Fig. 3A) or in a group (Fig. 3B) responding to 500 µM menthol. Menthol caused a Ca^2+^ rise in the midpiece and the lower part of the head that propagated towards the upper part of the head. This change was significantly inhibited by capsazepine (Fig. 3C and 3D). Representative traces of the menthol-induced and capsazepine inhibition of these responses are shown in the right panels of figure 3A, 3B and 3C, 3D, respectively. It is worth mentioning that only 40% of cells showed a [Ca^2+^]i rise upon menthol addition during the time of the experiment and capsazepine reduced both, the magnitude of the response (right panels 3A vs 3C and 3B vs 3D) and the number of responsive cells to about 15% (Supplementary Figure S3). Additionally, from menthol responsive cells, near half of them returned to basal levels and the rest showed a sustained increase during the time under examination.

**Figure 4:**
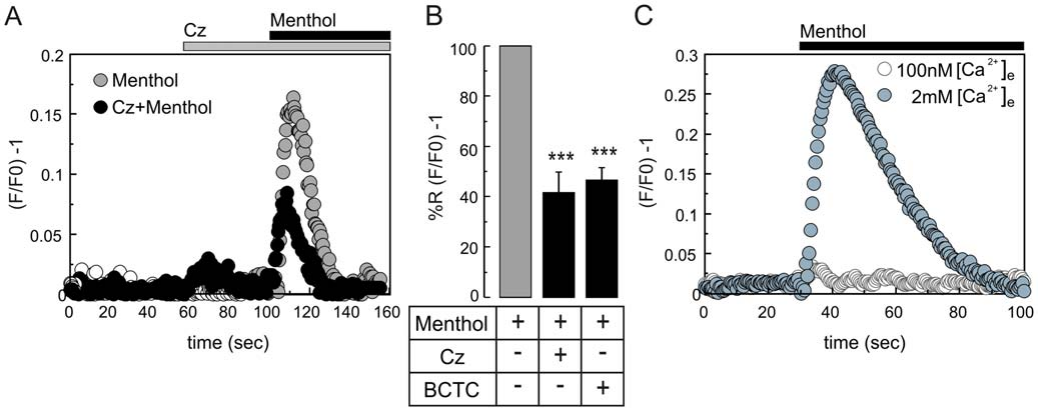
The menthol induced [Ca^2+^]i increase in human sperm populations is sensitive to capsazepine and BCTC and depends on external Ca^2+^. Human sperm were loaded with Fluo3-AM (2 µM) and the fluorescence intensity measured in cell population before and after menthol addition as described in methods. (A) Representative traces showing the fluorescence change after addition of 500 µM menthol (gray circles), this increase was partially blocked by capsazepine (Cz, 20 µM) (black circles), the duration of the stimulus is indicated by the bars above the graph. (B) Summary of the menthol response inhibition caused by 20 µM Cz or 1.6 µM BCTC (% relative fluorescence normalized to the fluorescence obtained after the addition menthol). (C) Representative menthol responses to 500 µM menthol in media containing 100 nM (open circles) or 2 mM (closed circles) external [Ca^2+^] ([Ca^2+^]_e_), the duration of menthol application is indicated by the black bar (n = 6, *** p< = 0.001).

The menthol induced [Ca^2+^]i rise was corroborated in sperm population experiments. Figure 4A shows representative traces of the increase in [Ca^2+^]i caused by 500 µM menthol in sperm suspensions incubated in the absence or presence of capsazepine. The increase was dose-dependent with an IC_50_∼250 µM (not shown). The menthol induced [Ca^2+^]i increase observed in these experiments was always transient and recovered to basal levels after about 40 seconds. Consistent with the AR inhibition observed with capsazepine, this TRPM8 antagonist blocked 60% of the [Ca^2+^]i rise; similar results were obtained with BCTC (Fig. 4B). Menthol can also increase [Ca^2+^]i independently of TRPM8 due to release from intracellular stores [13]. To distinguish between these possibilities, menthol responses were evaluated in conditions where external Ca^2+^ concentrations were lowered until reaching those found intracellularly (∼100 nM). The results showed that under this condition the signal was completely abolished (Fig. 4C), thus confirming the participation of TRPM8 in the menthol induced Ca^2+^ increase. In all experiments where inhibitors and/or menthol were used, the functional state of sperm was tested by measuring the progesterone-induced [Ca^2+^]i increase (not shown).

**Figure 5:**
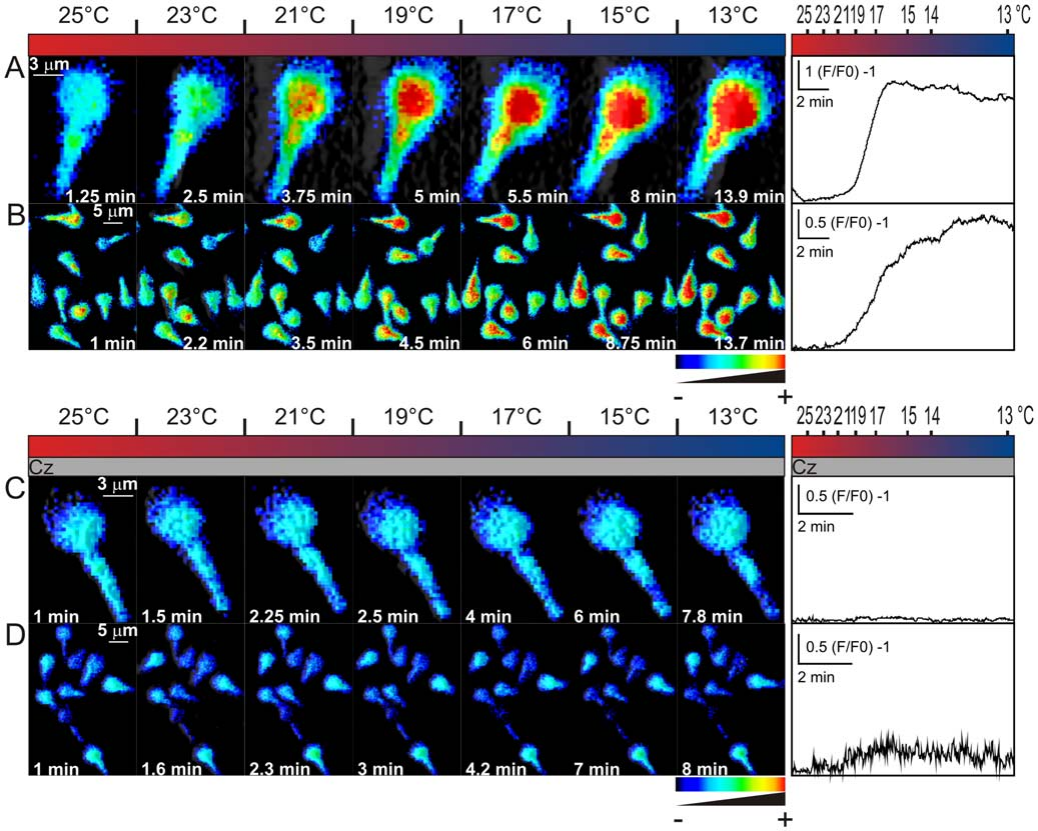
Lowering temperature from 25 to 13°C increases [Ca^2+^]i in human sperm. [Ca^2+^]i was monitored after loading cells with Fluo3-AM (2 µM) (see methods). Single cell (A and C) and group of cells (B and D) spatio-temporal [Ca^2+^]i changes induced by cooling (25–13°C) (red to blue bar) in the absence (A and B) or presence (C and D) of 20 µM Cz (gray bar). The panels to the right illustrate representative [Ca^2+^]i traces of the corresponding field during the temperature ramp in the absence (A and B) or presence (C and D) of 20 µM Cz. The time frame is indicated in each panel. Scales indicate (F/F0) -1 vs time (sec). Note: about 28% of cells responded to menthol. Color coding: black (−) to red (+) indicates low to high [Ca^2+^]. n≥3.

It is well known that temperature is the universal agonist of TRPM8, varying within the 18°C–25^o^C range, depending on cell type. Figure 5 shows individual (A and C) and group sperm [Ca^2+^]i responses (B and D) to a decreasing temperature ramp from 25°C to 13°C. A temperature dependent [Ca^2+^]i increase was clearly observed between 23°C–21°C, which was blocked by capsazepine (Figure 5C and 5D). The Q10 value calculated for the [Ca^2+^]i change was above 20 (not shown), confirming the temperature dependence of this process. Representative traces of the temperature-induced [Ca^2+^]i responses and their inhibition by capsazepine are shown in figures 5A, 5B and 5C, 5D, respectively. These results add a further support for the functional presence of TRPM8 channels in human sperm.

**Figure 6:**
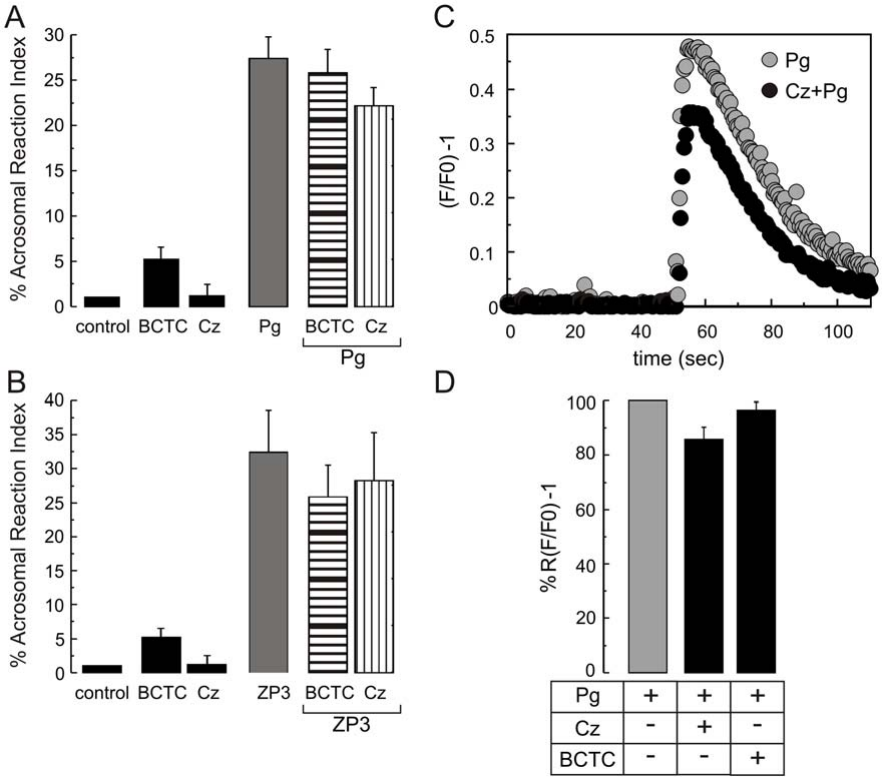
TRPM8 antagonists do not inhibit the human sperm AR induced by ZP3 or progesterone (Pg). Sperm were capacitated for ≥5 hours (see Methods) and the AR induced with 4 µM Pg (A) or recombinant human ZP3 (B) in the presence or absence of BCTC (1.6 µM) or Cz (20 µM). The ZP3 or Pg induced AR was insensitive to both inhibitors. Controls using the inhibitors alone are also shown (black bars). (C) Representative [Ca^2+^]i changes monitored after loading cells with Fluo3-AM in cell population experiments. The presence of capsazepine (black circles) did not significantly inhibit the progesterone (gray circles) induced [Ca^2+^]i increase. Scales indicate (F/F0) -1 vs time (sec). (D) Summary of the progesterone response in the absence (gray bar) or presence of 20 µM Cz or 1.6 µM BCTC (black bars), (% relative fluorescence normalized to the fluorescence obtained after the addition progesterone; n≥5).

Considering that TRPM8 stimulation induces [Ca^2+^]i increases and the AR in sperm, it seemed worth asking if this channel participates in the ZP or progesterone induced AR. For this purpose, recombinant human ZP3 (10 ng/µl) and progesterone (4 µM) were used to trigger the AR and BCTC and capsazepine were tested for their ability to inhibit the AR in these conditions. Figure 6 shows that BCTC and capsazepine neither affect the progesterone (Fig. 6A) nor the ZP3 (Fig. 6B) induced-AR. Cell population experiments corroborated these results since progesterone induced [Ca^2+^]i increase was also insensitive to capsazepine (Fig. 6C, D); similar results were obtained with BCTC (Fig. 6D). These results indicate that TRPM8 does not play a significant role in ZP3- or progesterone-driven AR.

## Discussion

The TRP superfamily of channels is large and functionally very versatile. These channels participate in sensory functions such as vision, taste and olfaction. Individual cells use these channels to sense temperature, osmolarity, pH, humidity, etc [6], [14]. Sperm encounter a wide variety of environmental cues while searching for the egg along the female genital tract. Although the presence of several members of the TRPC subfamily in mammalian sperm has been reported and their role on swimming, capacitation and the AR is being studied, [3], [5], [15] very little is known about the presence and physiological importance of other TRP subfamilies in sperm.

TRPM8 was initially identified in prostate and found to be up-regulated in prostate cancer; it was thought to be a prostate specific gene. Later TRPM8 was recognized as a thermo sensitive channel and detected in trigeminal neurons and sensory neurons of the dorsal root ganglia. However, it is now know that TRPM8 has a wider tissue distribution and is present in non-neuronal cells and tissues that usually do not experience low temperatures such as gut [16], vascular smooth muscle [17], liver [18] and lung [19], therefore its role is far from being understood. TRPM8 has been also proposed to function as an intracellular channel in certain cell types [20].

In this study, we explored the presence of TRPM8 channels in human sperm. Interestingly, we found TRPM8 transcripts and expression of the protein in the sperm flagella and head. Considering its location in the flagella, the influence of menthol on motility was examined. This agonist did not alter the gross motility parameters of sperm determined by CASA. Although CASA analysis has been proven useful for several applications, a detailed analysis of flagellar movements cannot be performed under physiological conditions. A more sophisticated single cell study is needed to fully explore the participation of TRPM8 in motility regulation. This is relevant considering recent evidence indicating that mammalian sperm exhibit both thermo and chemo taxis and that they are important for mammalian fertilization.

How sperm sense temperature and chemical gradients is not fully understood. Several G-coupled olfactory receptors have been identified in sperm but their physiological agonists remain unknown [7]. Multiple chemotactic and sensory processes may be required for sperm to reach the egg [21]. This could explain why these cells need a large channel network to do the job. The nature of the egg's chemoattractant(s) is not yet clear but one proposed candidate is progesterone. It has been suggested that chemoattraction could only take place in a short segment of the fallopian tube, since the peristaltic movement in this tissue would disrupt a chemoattractant gradient in a long stretch [22]. Therefore, sperm would be first guided to the egg by a thermotactic mechanism during a long section of the female tract and only the final stretch would use chemoattraction. Thermotaxis has been documented in human and rabbit sperm, and only capacitated sperm are thermo-guided [23]. It is tempting to speculate that TRPM8, a temperature-sensitive channel, may have a role during thermotaxis. As shown in this work, a decrease from 23 to 18°C induced a [Ca^2+^]i increase in about 30% of sperm and capsazepine reduced both, the magnitude of the response and the number of responsive cells.

The temperature threshold reported for recombinant TRPM8 covers a wide range of temperatures and differs from native channels. Molecular determinants of threshold differences among cold receptors are unknown and under investigation. Several studies document TRPM8 modulation by a variety of stimulus demonstrating an important flexibility in the temperature response curve of TRPM8 channels which can vary by more than 15°C [24]. Examples of TRPM8 modulators are: phosphoinositides [25], PIP2 [26]–[28], phosphorylation [28], [29], inorganic polyphosphate [30] channel density, intracellular Ca^2+^ levels [27], the variable expression ratio of K^+^/TRPM8 channels [31], [32] and lipid rafts [33].

It is well established that during sperm capacitation, cholesterol is lost from the sperm plasma membrane, a condition that certainly influences lipid rafts. Therefore, these maturational changes that occur in the female genital tract may shift the temperature threshold for TRPM8 to warmer temperatures [33]. In summary, we are just beginning to understand how temperature sensitive channels are regulated, and it is possible that in sperm, these channels are also subject to multiple forms of modulation. With all this in mind, sperm TRPM8 channels may be activated by temperature changes within the range encountered in the female tract.

The work presented here initiates the study of a thermo-sensitive channel found in sperm, but several other members of the TRP superfamily remain to be studied. A subset of these channels may be important for sperm guidance and for other sperm functions. The deorphanization of TRP channels is advancing and new pharmacological tools are being developed to unravel their participation in signaling [34]. Such tools together with novel sensitive imaging strategies to determine flagellar beating frequency, bending angle, [Ca^2+^]i, [35]–[37] and tridimensional sperm swimming [38], are opening new possibilities to explore how TRPs participate in sperm guidance.

Notably, addition of menthol caused a [Ca^2+^]i increase leading to AR. As anticipated, this process was inhibited by two TRPM8 antagonists: BCTC and capsazepine. About 70% of human sperm underwent menthol-induced AR, consistent with ∼50% of sperm showing menthol induced [Ca^2+^]i increases in single cell experiments. Interestingly, this lack of response in a subset of cells was also reported in human glioblastoma cells in which only between 14–58% showed a [Ca^2+^]i rise upon menthol addition [39]. The reason for this lack of reactivity is unknown. In our case, it is worth mentioning that sperm populations are highly heterogeneous, possibly reflecting the complex maturational processes needed for fertilization during transit along the epididymus and the female reproductive tract [40].

As in other exocytotic processes, [Ca^2+^]i and membrane fusion are intimately related in the sperm AR. During this process, the outer acrosomal membrane fuses with the inner plasma membrane releasing the acrosomal content (mainly hydrolytic enzymes) that helps sperm penetrate the extracellular egg's matrix and reach the plasma membrane. The egg's extracellular coat is composed of several glycoproteins; human eggs express ZP1, ZP2, ZP3 and ZP4. Most current models consider that ZP3 is the physiological AR inducer and that ZP3 binding to its sperm receptor(s) (not yet fully characterized) produces a biphasic rise in [Ca^2+^]i. This Ca^2+^ entry is essential for AR completion. Artificially raising [Ca^2+^]i with ionophores can trigger AR. At least three different Ca^2+^ channels have been implicated in the ZP3-induced AR. The pharmacology and kinetics of the first, fast (∼200 ms) [Ca^2+^]i transient are consistent with opening of Ca_v_ channels (Voltage dependent Calcium Channels) [41]. Ca_v_ channels usually require a depolarization to open. Though the identity of the depolarizing channel is still unknown, a Cl^−^ channel such as the Glycine receptor has been a proposed candidate [42]. Alternatively, TRPM8 could play this role in mammalian sperm. For instance, in a subpopulation of primary afferent neurons TRPM8 has been proposed to induce the depolarization that triggers an action potential [43]–[45]. ZP3 binding to sperm also causes the activation of a Ca^2+^-sensitive PLC and IP3 and diacylglycerol production. IP3 then binds to its receptor (IP3R, second channel) located in the acrosome [46], [47] releasing Ca^2+^ from this intracellular store which activates a plasma membrane Store Operated Channel (SOC) (third channel) leading to a sustained [Ca^2+^]i increase lasting several minutes. These SOCs may be constituted by TRP family members [3], [15].

The signaling model described is based mainly on results from mouse sperm, but a similar sequence of events has been proposed for the human AR [48]. Due to the lack of native human ZP3, progesterone has been widely used to study the human sperm AR. Though progesterone also causes a biphasic [Ca^2+^]i increase, its signaling cascade is different from that of ZP3 [1]. TRPM8 activation significantly increases [Ca^2+^]i and also induces the AR; however, the pharmacology of these responses differs from that of the physiological inducers, indicating a distinct signaling cascade. In agreement with this notion, TRPM8 null mice are fertile [49]. Therefore, the physiological role of TRPM8 in the mouse sperm AR remains to be established. It could participate in the ill defined ZP4-induced AR [50], in alternate paths leading to this reaction needed under altered physiological conditions or to decrease its threshold, as has been postulated for progesterone [51].

Finally, TRP channels integrate multiple signals such as temperature, osmolarity, pH changes, mechanical stress, etc. Their heteromultimeric nature contributes to their diverse regulation [52]; thus identification of the subunit composition of the functional entity in a particular cell type is essential to understand its physiological role.

## Supporting Information

Figure S1 Menthol does not influence human sperm motility. Human sperm separated by swim up were tracked and analyzed with the Hobson Tracker computer-aided semen analysis system. Thirty frames were acquired at a frame rate of 60 Hz. The following parameters are shown: % of motile sperm (%Mot), % of sperm with active motility (%Act), % of sperm with hyperactivated motility (%Hyper), amplitude of lateral head displacement (ALH, μm), curvilinear velocity (VCL, μm/s), and the derived parameters of linearity (LIN, %) and straightness (STR, %). Sperm were exposed to up to 1 mM menthol (white bars) and none of the parameters were significantly different from the control (black bars) in non capacitating (top panel) or capacitating (bottom panel) conditions(n = 7).
(0.81 MB TIF)

Figure S2 Positive control for the TRPM8 antibody. Total protein homogenates from human sperm and mouse brain were subjected to Western Blot experiments with anti-TRPM8 (Santa Cruz Biotechnology) and a band of the appropriate molecular weight (arrow head) was detected in both samples. The additional bands of lower molecular weight probably represent degradation products of the same protein.
(0.09 MB TIF)

Figure S3 Capsazepine reduces the number of menthol and temperature responsive cells. Percentage of sperm undergoing [Ca^2+^]i responses when exposed to menthol (500 µM) (A) or decreasing temperature (B) in the absence (black) or presence (gray) of 20 µM Cz. This antagonist not only diminishes the magnitude of the responses (Fig. 3C, 3D and 5C, 5D) but also decreases the number of responsive cells. n≥3, at least 200 hundred cells were evaluated per condition.
(0.48 MB TIF)
