# Supplementary figures and images for: TRPM8, a Versatile Channel in Human Sperm
Source: PLoS One. 2009 Jun 30;4(6):e6095. doi: 10.1371/journal.pone.0006095 (PMC2705237; doi:10.1371/journal.pone.0006095)

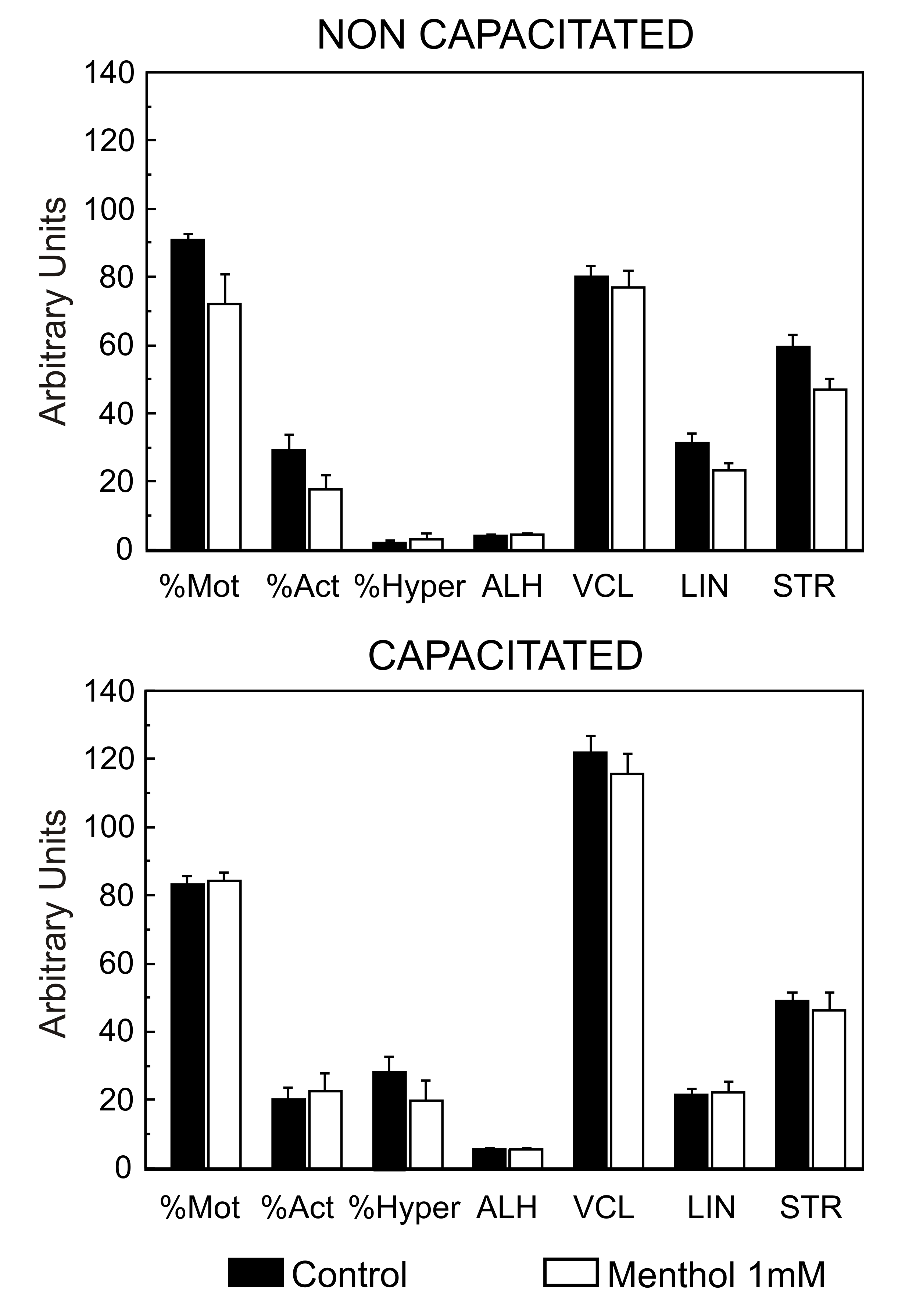

Supplement: Figure S1 — Menthol does not influence human sperm motility. Human sperm separated by swim up were tracked and analyzed with the Hobson Tracker computer-aided semen analysis system. Thirty frames were acquired at a frame rate of 60 Hz. The following parameters are shown: % of motile sperm (%Mot), % of sperm with active motility (%Act), % of sperm with hyperactivated motility (%Hyper), amplitude of lateral head displacement (ALH, μm), curvilinear velocity (VCL, μm/s), and the derived parameters of linearity (LIN, %) and straightness (STR, %). Sperm were exposed to up to 1 mM menthol (white bars) and none of the parameters were significantly different from the control (black bars) in non capacitating (top panel) or capacitating (bottom panel) conditions(n = 7). (0.81 MB TIF) [file pone.0006095.s001.tif]

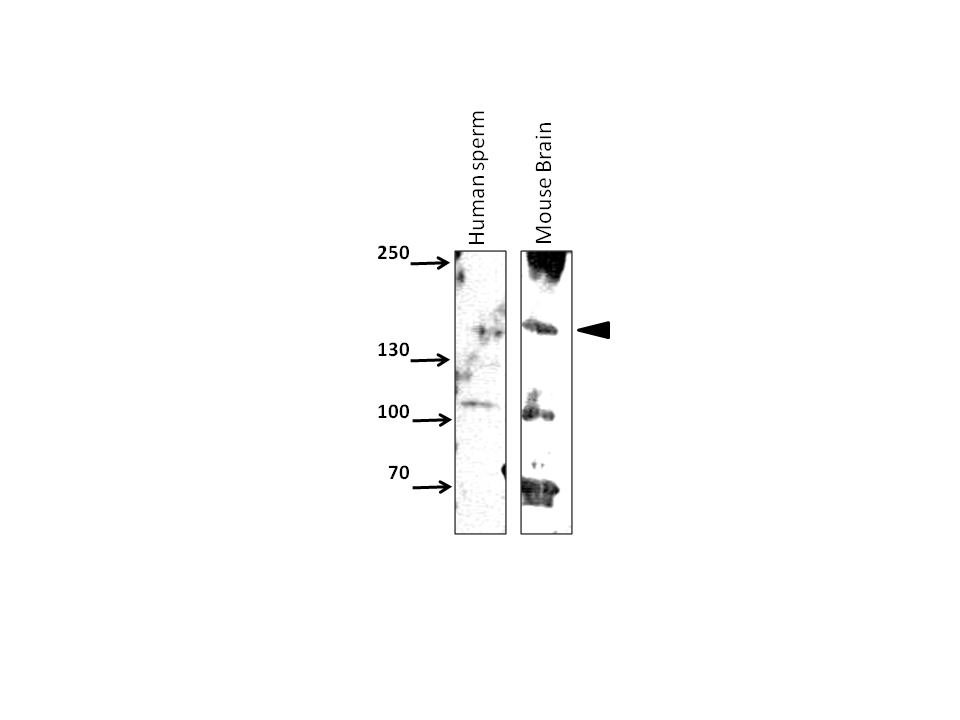

Supplement: Figure S2 — Positive control for the TRPM8 antibody. Total protein homogenates from human sperm and mouse brain were subjected to Western Blot experiments with anti-TRPM8 (Santa Cruz Biotechnology) and a band of the appropriate molecular weight (arrow head) was detected in both samples. The additional bands of lower molecular weight probably represent degradation products of the same protein. (0.09 MB TIF) [file pone.0006095.s002.tif]

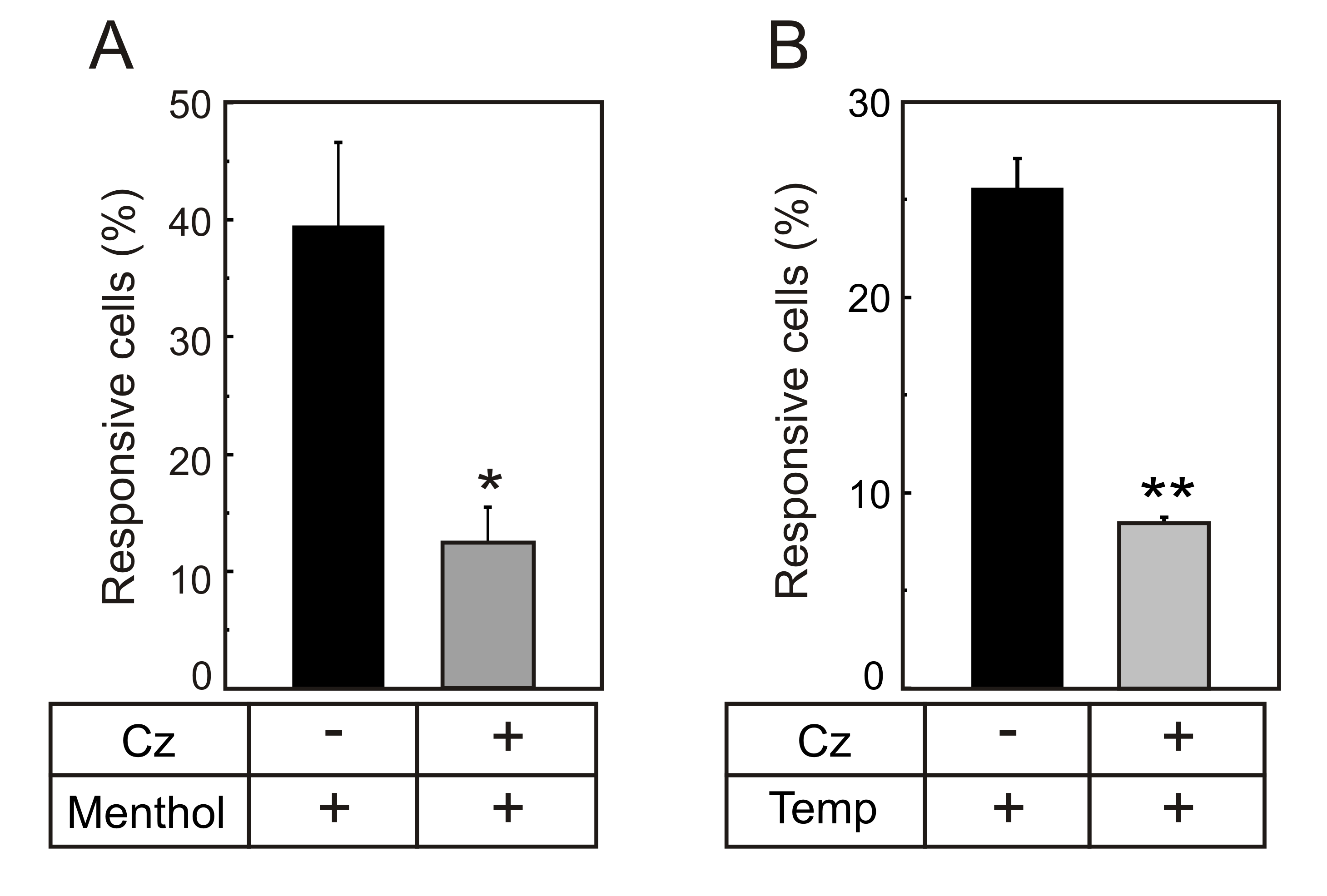

Supplement: Figure S3 — Capsazepine reduces the number of menthol and temperature responsive cells. Percentage of sperm undergoing [Ca2+]i responses when exposed to menthol (500 µM) (A) or decreasing temperature (B) in the absence (black) or presence (gray) of 20 µM Cz. This antagonist not only diminishes the magnitude of the responses (Fig. 3C, 3D and 5C, 5D) but also decreases the number of responsive cells. n≥3, at least 200 hundred cells were evaluated per condition. (0.48 MB TIF) [file pone.0006095.s003.tif]
